# Extracellular Signal-Regulated Kinases Mediate an Autoregulation of GABA_B_-Receptor-Activated Whole-Cell Current in Locus Coeruleus Neurons

**DOI:** 10.1038/s41598-020-64292-x

**Published:** 2020-05-12

**Authors:** Rui-Ni Wu, Chao-Cheng Kuo, Ming-Yuan Min, Ruei-Feng Chen, Hsiu-Wen Yang

**Affiliations:** 1 Department of Life Science, and National Taiwan University, Taipei, 106 Taiwan; 2 Center for Neurobiology and Cognition Science, National Taiwan University, Taipei, 106 Taiwan; 3 Department of Biomedical Sciences, Chung Shan Medical University, Taichung, 402 Taiwan; 4 Department of Medical Research, Chung Shan Medical University Hospital, Taichung, 402 Taiwan

**Keywords:** Cellular neuroscience, Neurophysiology

## Abstract

The norepinephrine-releasing neurons in the locus coeruleus (LC) are well known to regulate wakefulness/arousal. They display active firing during wakefulness and a decreased discharge rate during sleep. We have previously reported that LC neurons express large numbers of GABA_B_ receptors (GABA_B_Rs) located at peri-/extrasynaptic sites and are subject to tonic inhibition due to the continuous activation of GABA_B_Rs by ambient GABA, which is significantly higher during sleep than during wakefulness. In this study, we further showed using western blot analysis that the activation of GABA_B_Rs with baclofen could increase the level of phosphorylated extracellular signal-regulated kinase 1 (ERK_1_) in LC tissue. Recordings from LC neurons in brain slices showed that the inhibition of ERK_1/2_ with U0126 and FR180204 accelerated the decay of whole-cell membrane current induced by prolonged baclofen application. In addition, the inhibition of ERK_1/2_ also increased spontaneous firing and reduced tonic inhibition of LC neurons after prolonged exposure to baclofen. These results suggest a new role of GABA_B_Rs in mediating ERK_1_-dependent autoregulation of the stability of GABA_B_R-activated whole-cell current, in addition to its well-known effect on gated potassium channels, to cause a tonic current in LC neurons.

## Introduction

γ-Aminobutyric acid (GABA) is the principal inhibitory neurotransmitter in the forebrain. By acting at ionotropic GABA_A_ receptors (GABA_A_Rs) located within the synaptic acting zone, GABA can rapidly increase the membrane permeability to Cl^-^ in target neurons and produce fast phasic inhibitory transmission. This type of signaling is referred to as conventional synaptic transmission and features a specific method of communication between neurons with high temporal and spatial precision that enables the presynaptic neuron to shape the spiking pattern of the postsynaptic neuron. In addition to those located in the synaptic active zone, GABA_A_Rs containing specific subunits can also mediate a tonic form of inhibition that is not time-locked to presynaptic action potentials (APs) and is shown to profoundly modulate the input–output relationships of individual neurons. GABA_A_R-mediated tonic inhibition has been identified as an important player in both physiological and pathophysiological processes^1,2^.

In addition to GABA_A_Rs, GABA also acts on metabotropic GABA_B_ receptors (GABA_B_Rs) to produce a much slower but very long-lasting inhibition at both presynaptic and postsynaptic sites^3–7^ compared with the fast phasic transmission mediated by GABA_A_Rs. At the presynaptic site, the activation of GABA_B_Rs reduces the release probability of synaptic vesicles through inhibiting N-type or P/Q-type voltage-dependent Ca^2+^channels; at the postsynaptic site, the activation of GABA_B_Rs produces hyperpolarization by increasing the potassium conductance of G protein-coupled inwardly rectifying K^+^(GIRK) or inwardly rectifying K^+^3 (Kir3) channels^8–10^. GABA_B_Rs were the first G protein-coupled receptor (GPCR) to be identified as an obligate heterodimer; a functional GABA_B_ receptor is formed from the heterodimerization of the GABA_B1_ and GABA_B2_ receptor subunits, with the former constituting the GABA binding site and the latter being coupled to the Gproteins, comprising α_i/o_, β and γ subunits^11–13^. The binding of GABA to the GABA_B1_ receptor activates the coupled G protein to gate the pre- and postsynaptic ion channels described above via the β and γ subunits^8,10^. Despite the well-understood functional roles of the β and γ subunits, much remains to be learned about the role of receptor-induced lowering of cAMP levels by the α_i/o_ subunit.

Electron microscopic studies have revealed that the subcellular distribution of GABA_B_Rs is mostly at peri-/extrasynaptic loci^4–7^, implying that, similar to GABA_A_Rs, these extrasynaptic GABA_B_Rs can mediate a tonic form of signaling by detecting ambient GABA. Indeed, it has been shown that ambient GABA can tonically induce a low level of presynaptic and postsynaptic GABA_B_R activation to provide the control of transmitter release at the hippocampus and calyx of Held synapses and the control of the excitability of pyramidal neurons in the medial prefrontal cortex and noradrenergic (NAergic) neurons in the locus coeruleus (LC)^7,9,14–16^. The physiological roles of GABA_B_R-mediated tonic inhibition have begun to emerge. Recently, it has been shown that tonic inhibition of LC NAergic neurons (hereafter referred to as LC neurons) could be an important player in the regulation of brain function states^7,17^. LC neurons have global NAergic projections to the forebrain and play important roles in the control of behaviors through the regulation of vigilance^18,19^. Furthermore, GABAergic transmission in the LC has been implied to be a mechanism underlying the effect of some anesthetics on consciousness^17,20–24^. It has been shown that LC neurons and NAergic A7 neurons in the pons express a large amount of GABA_B_Rs and are subject to GABA_B_R-mediated tonic inhibition in brain slice preparations and *in vivo*^7,9,17^. Moreover, the suppression of the tonic inhibition of LC neurons could accelerate the regain of consciousness from isoflurane-induced deep anesthesia^17^. Tonic inhibition would require the activity of a substantial number of GABA_B_Rs on the membrane for a long period. Nevertheless, this would appear to conflict with the features of most GPCRs, including GABA_B_Rs, such that the receptor will undergo rapid desensitization upon activation by the ligand^25^. In this study, we report that, in LC neurons, the activation of GABA_B_Rs also activates extracellular signal-regulated kinase 1 (ERK_1_) signaling pathways, which is consistent with previous studies in the hippocampus and cerebellum^26–28^. We further show that the activation of ERK_1_ signaling pathways by GABA_B_Rs could prevent a rapid decline in the GABA_B_R-activated whole-cell membrane current and help stabilize tonic inhibition.

## Results

### Activation of GABA_B_Rs increases pERK levels in the LC

**Figure 1:**
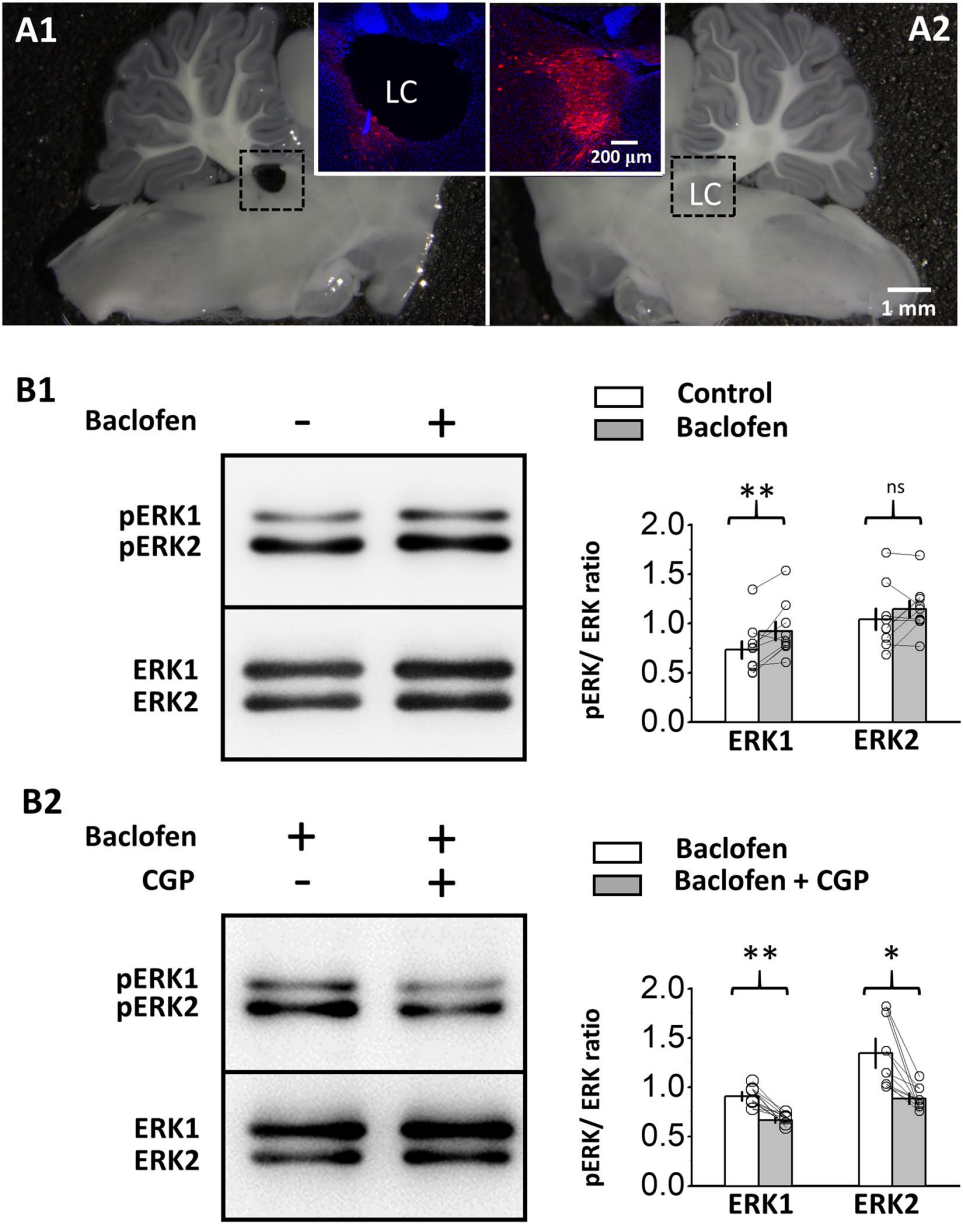
The activation of GABA_B_Rs increases pERK_1_ levels in LC tissue. (**A**) The images show two sagittal brainstem slices from an animal. The LC in the left slice was punched (A1) for western blot analysis, and the right slice was used for comparison (A2). IHC with anti-TH antibody was performed for the two slices, as shown in the insets showing merged fluorescence images of anti-TH (red) and DAPI (blue) staining of the dashed rectangular areas at high magnification. A comparison of the two slices shows that the punched area contained mostly TH-ir tissue. (**B**) Images show representative western blot analysis results for pERK_1/2_ in LC tissue punched from slices bathed in vehicle or baclofen (B1) and from slices bathed in baclofen or baclofen plus CGP54626 (B2). The plot in the right panels summarizes the results. Each paired circle and line indicates the result of a single experiment; bars and capped lines denote the mean and SEM, respectively. The asterisks denote significant differences compared to the control at p < 0.05 (*) and p < 0.01 (**); ns denotes no significance compared to the control.

We first examined whether the activation of GABA_B_Rs could also increase phosphorylated-ERK_1/2_ (pERK_1/2_) levels in the LC, as previously reported in hippocampal and cerebellar tissue^26–28^. We examined pERK_1/2_ levels in LC tissue punched from slices (Fig. 1A) bathed in 50 μM baclofen, a GABA_B_R agonist, and the vehicle, artificial cerebrospinal fluid (aCSF) containing synaptic blockers (see Materials and Methods), using western blot analysis. As pERK_1/2_ levels were reported to peak at 10 min and start to decline at 20 min of baclofen stimulation in cultured cerebellar granule cells^27^, 15 min of baclofen stimulation was used in this study. In comparison to the tissues from the vehicle-bathed slices, the pERK_1_ level increased by 29.5 ± 8.2% in the LC tissues from the baclofen-bathed slices (Fig. 1B1) (p = 0.006; n = 9, Student’s paired t-test). There was no increase in the pERK_2_ level (p = 0.183; n = 9, Student’s paired t-test). We also compared pERK_1/2_ levels between LC tissues punched from slices bathed in baclofen and in baclofen plus 10 μM CGP54626, a GABA_B_R antagonist. Compared to LC tissues from the baclofen-bathed slices, the inhibition of GABA_B_Rs with CGP54626 reduced the pERK_1_ level by 25.9 ± 4.1% (Fig. 1B2) (p = 0.003, n = 6, Student’s paired t-test), showing that the increase in the pERK_1_ level by baclofen stimulation was specific to GABA_B_R activation. Interestingly, compared to LC tissues from the baclofen-bathed slices, the pERK_2_ level also significantly decreased by 31.3 ± 6.1% in CGP54626-bathed slices (p = 0.018, n = 6, Student’s paired t-test). As the ambient GABA in the pontine area can continuously activate GABA_B_Rs to exert tonic inhibition of LC neurons^7,9^, it could be that there was a basal pERK_2_ level produced by continuous GABA_B_R activation. Accordingly, tonic GABA_B_R activation left less room for a further increase in the pERK_2_ level by baclofen stimulation, and the inhibition of GABA_B_Rs could result in a significant reduction.

### Characterization of I_GABABR_ in LC neurons

**Figure 2:**
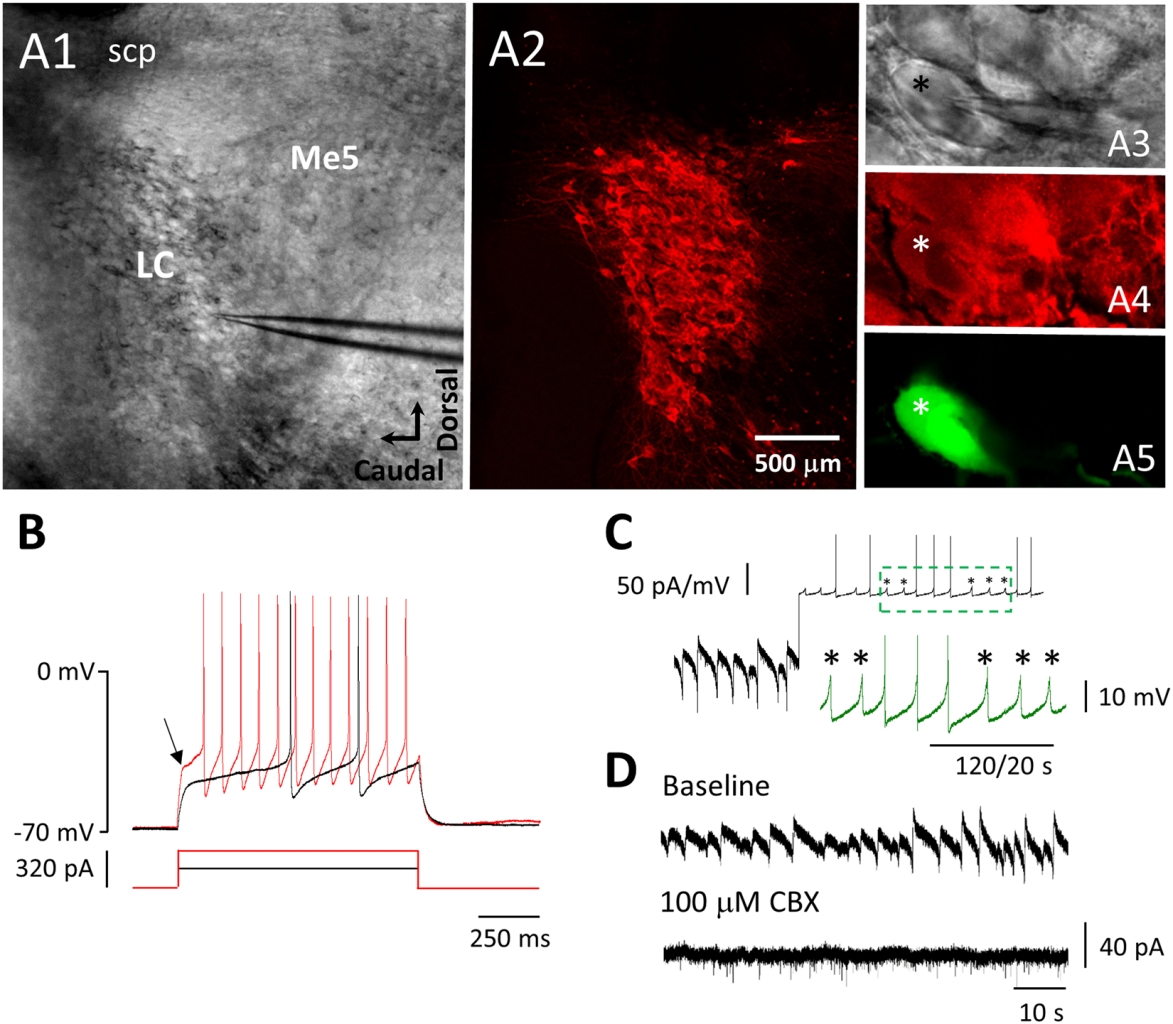
Recordings from LC neurons (**A**) Images showing the identification of LC neurons with post hoc IHC using the anti-TH antibody. A1 and A3 show online phase contrast images of a sagittal brainstem slice at low (A1) and high magnification (A3). A2 and A4 show fluorescence images of anti-TH staining of the same field and magnification as shown in A1 and A3, respectively. A5 shows a fluorescence image of the same field and magnification as in A4 showing a recorded neuron filled with biocytin. This neuron also displayed TH-ir, as indicated by the asterisk. Abbreviations: **Me5**, mesencephalic trigeminal nucleus; **scp**, superior cerebellar peduncle. (**B**) Representative current-clamp recording from the TH-ir (LC) neuron shown in A, showing V_m_ responses (top traces) to current injection (bottom traces). Note the delay in the onset of AP (see arrow) elicited from V_m_ held at −70 mV. (**C**) A representative V-clamp (left bottom half) and I-clamp (right upper half) recording from an LC neuron. The arrow indicates switching of the recording from V-clamp to I-clamp mode. Note the biphasic I_Osc_ in the V-clamp recording and the spontaneous APs and voltage oscillation in the I-clamp recording. The inserted green trace shows activity marked by the dashed rectangle on a faster and larger scale. Asterisks mark the voltage oscillations. The vertical bar to the left of the trace shows the amplitude scale for V-clamp (50 pA) or I-clamp (50 mV) recordings; the one to the right of the inserted trace shows the amplitude scale for the inserted trace; the bottom horizontal bar shows the time scale for the whole trace (120 s) and the inserted trace (20 s). (**D**) A representative experiment with V-clamp recordings from an LC neuron showing that I_Osc_ are blocked by the application of 100 μM CBX, a gap junction blocker; top and bottom traces show recordings before and after CBX application, respectively.

To confirm that the increase in pERK_1_ levels upon GABA_B_R activation occurred in LC neurons and to explore the possible physiological role of the elevated ERK activity, we performed whole-cell patch recording from LC neurons and tested the effects of ERK blockers on the whole-cell current induced by GABA_B_R activation. All recordings described hereafter were performed with the addition of synaptic blockers to the bath medium to avoid secondary effects via fast synaptic transmissions. We adapted previously described criteria for identifying NAergic neurons in the dorsal pontine area^7,29,30^ to validate that the recorded neurons were LC neurons. The criteria were as follows: (1) the recorded neuron should be immunoreactive to anti-tyrosine hydroxylase (TH) antibody (Fig. 2A); (2) the recorded neuron should be able to spontaneously fire APs followed by prominent afterhyperpolarization; and (3) the recorded neuron should display a delay in AP generation upon the injection of depolarizing current pulses, with V_m_ held at ~ −70 mV (Fig. 2B). A very interesting observation from the whole-cell recordings of LC neurons was the appearance of spontaneous oscillation at ~ 0.2 Hz of the membrane voltage in current-clamp recordings (Fig. 2C) or of the membrane current in voltage-clamp recordings (Fig. 2D). The oscillating events displayed some similar features to those of spontaneous APs, such as being biphasic and generated at a rate similar to spontaneous APs. Since LC neurons are electrically coupled to gap junctions^31,32^, these events could be due to flow through the gap junctions of currents underlying the APs generated from other LC neurons in the proximity and electrically coupled to the recorded neuron^7^. This argument is further supported by the results showing that these events were blocked by CBX, a gap junction blocker (Fig. 2D). We refer to the events recorded using the voltage clamp as I_Osc_.

**Figure 3:**
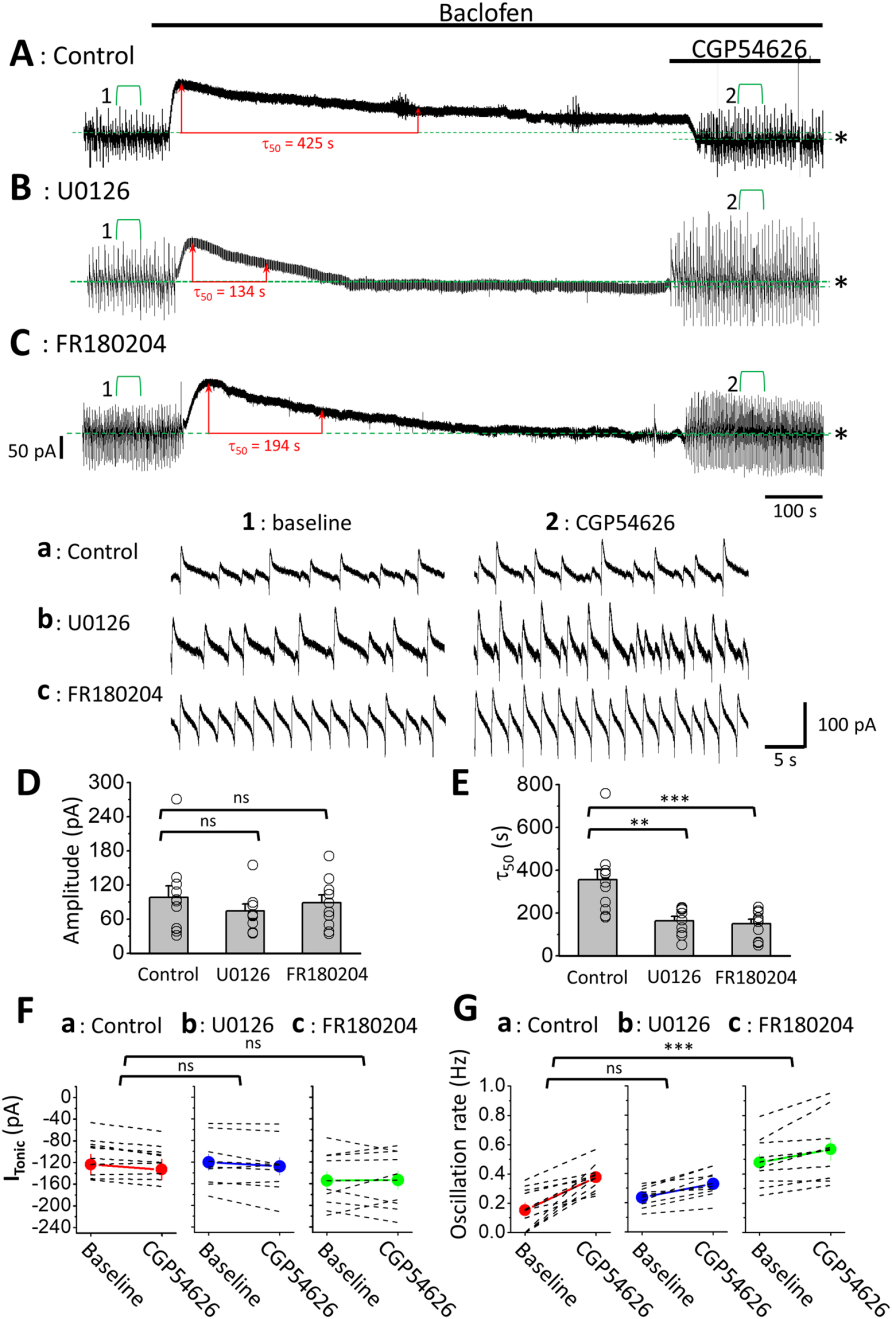
The inhibition of ERK_1/2_ decreases the τ_50_ of I_GABABR_ in LC neurons. (**A**-**C**) Representative recording of I_GABABR_ from LC neurons in the control slice (A), U0126-treated slice (B), and FR180204-treated slice (C). The red double-headed line marks τ50, and the long and short green dashed lines mark the means of the membrane current recorded at baseline (before baclofen) and upon CGP54626 application. Note that the difference is measured as I_Tonic,_ as indicated by the asterisk. The activity marked with the green square bracket is enlarged and shown at the bottom (traces a-c). Note the increased frequency of I_Osc_ with CGP54626 application compared with baseline. (**D**-**G**) Plots show summarized results of the amplitude (D) and τ_50_ (E) of I_GABABR_, I_Tonic_ (F) and the rate of I_Osc_ (G). Each circle (D-E) or dashed line (F-G) shows the result of an individual experiment; bars (D-E) or circles (F-G) denote the mean, and capped lines denote the SEM. The asterisks indicate a significant difference in τ_50_ (E) or in the increment of I_Osc_ frequency (G) compared to the control at p < 0.01 (**) or at p < 0.005 (***). p denotes a significant increase in I_Osc_ frequency after CGP54626 application (G); ns denotes no significant difference compared to the control.

Bath application of 100 μM baclofen induced an outward current that was blocked by subsequent application of 10 μM CGP54626 (Fig. 3A), showing that the current was mediated by GABA_B_Rs. This observation is consistent with our previous reports^7,9^, further demonstrating that the current was generated by the opening of GIRK channels downstream of the activation of GABA_B_Rs by baclofen. Hereafter, we refer to the current as the GABA_B_R-mediated current (I_GABABR_). Interestingly, the induction of I_GABABR_ was associated with the suppression of I_Osc_ activity, and the activity reappeared upon subsequent application of CGP54626 to counteract the effect of baclofen (Fig. 3A–C).

The I_GABABR_ underwent partial decline upon prolonged (15 minutes) exposure to baclofen in the control condition. As seen in Fig. 3A, upon the application of baclofen, I_GABABR_ was quickly induced and peaked with a mean amplitude of 98.1 ± 20.4 pA (n = 11; Fig. 3D), followed by a gradual decline to approximately half the peak amplitude. We quantified the decline in I_GABABR_ by measuring τ_50_, defined as the time required for the I_GABABR_ to decline from its peak amplitude to half that value, and it was 356 ± 49 s in the control condition (Fig. 3E). The subsequent coapplication of CGP54626 15 min after baclofen application suppressed I_GABABR_ to a level below baseline (the membrane current before baclofen application; see green dotted lines and asterisk in Fig. 3A), showing a basal tone of GABA_B_R activation in LC neurons. The membrane current underlying the basal tone of GABA_B_R activation is referred to as the tonic current (I_Tonic_), measured as the difference between the membrane currents at baseline and upon CGP54626 application (see asterisks in Fig. 3A). The I_Tonic_ in the control was −9.4 ± 2.4 pA (n = 11) (Fig. 3F). Consistent with the observation of the basal tone of GABA_B_R activation, we also observed a significantly higher frequency of I_Osc_ during CGP54626 application than at baseline.The frequency of I_Osc_ at baseline and during the CGP54626 application was 0.15 ± 0.05 Hz and 0.38 ± 0.03 Hz, respectively (n = 11; p = 0.001, post hoc Bonferroni test after two-way repeated-measures ANOVA) (Fig. 3Aa, Fig. 3G).

### Inhibition of ERK_1/2_ accelerates the decline of I_GABABR_ LC neurons

**Figure 4:**
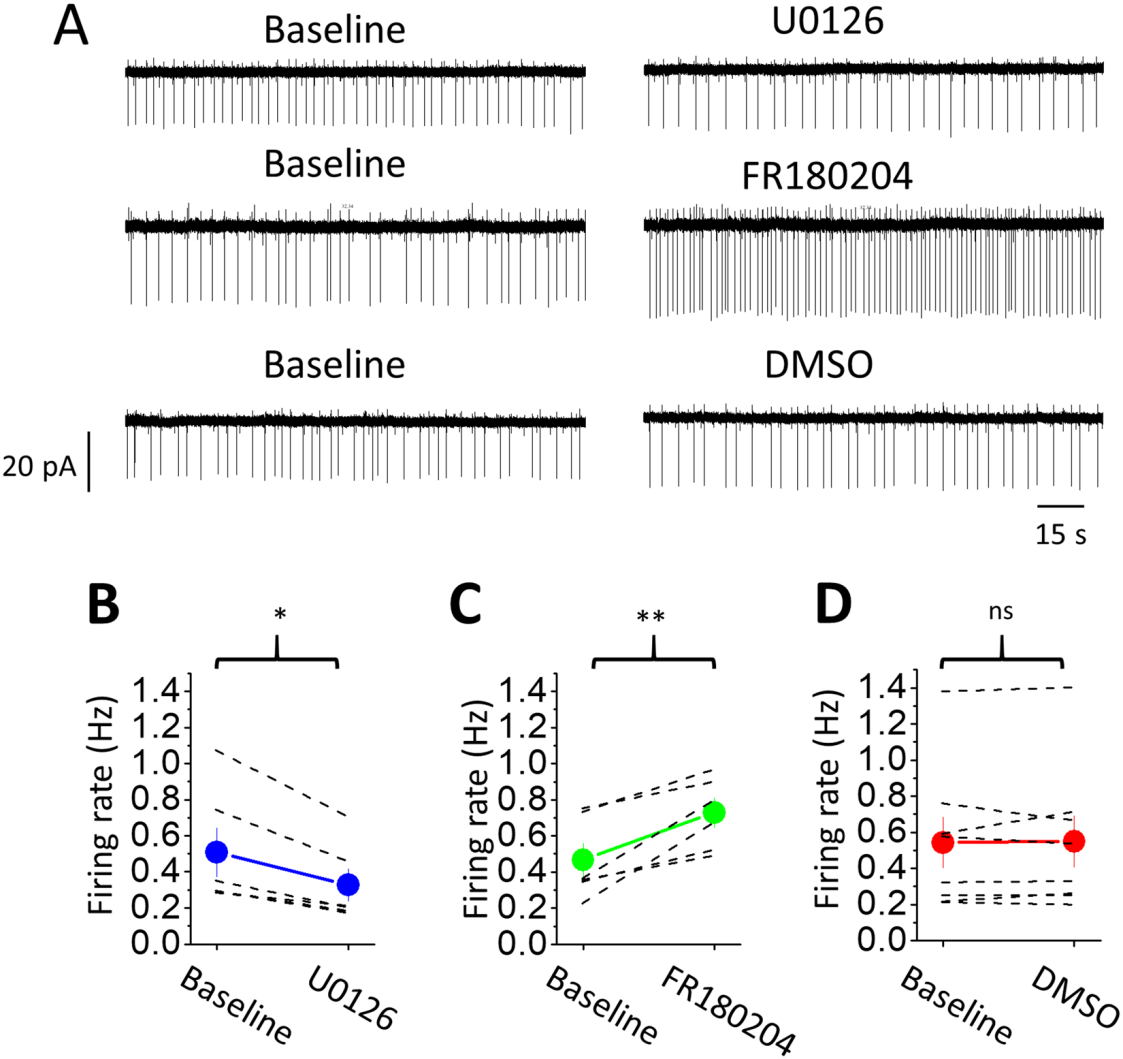
Effects of ERK_1/2_ inhibitors on the firing rate of LC neurons. (**A**) Representative episodes of firing rate recording from LC neurons before (baseline) (left panel) and after 30 mins of drug applications (right panel). The top, middle and bottom traces show the application of U0126, FR180204 and the vehicle (DMSO), respectively. (**B-D**) Summarized results show the change in the SFR upon U0126 (B), FR180204 (C) and DMSO (D) application. The asterisks indicate a significant difference compared to the control at p < 0.05 (*) or at p < 0.01 (**).

We next tested the effects of two selective ERK blockers, U0126 and FR180204, on I_GABABR_ in LC neurons. We first examined whether there were possible effects of the two ERK_1/2_ inhibitors on LC neurons by recording the basal spontaneous firing rate (SFR). The cell-attached configuration of the patch recording was used to avoid interference in the ion composition of the cytoplasm by the pipette solution. We found that the application of 20 μM U0126 for 30 min significantly decreased the SFR from 0.51 ± 0.13 to 0.33 ± 0.09 Hz (Fig. 4A,B) (n = 6 cells, p = 0.028, paired Wilcoxon-sign rank test). In contrast, the application of 20 μΜ FR180204 for 30 min significantly increased the SFR from 0.47 ± 0.09 to 0.73 ± 0.08 Hz (Fig. 4A,C) (n = 6 cells, p = 0.006, Student’s paired t-test), and the application of the vehicle (0.1% DMSO) did not have an effect on the SFR (Fig. 4A,D) (baseline: 0.54 ± 0.14 Hz, DMSO: 0.55 ± 0.14 Hz, n = 8 cells, p = 0.8, Student’s paired t-test). The differential effects of the two drugs on the SFR might be ascribed to the fact that FR180204 directly targets ERK_1/2_, while U0126 targets the mitogen-activated protein kinase that acts upstream of ERK_1/2_. The results also suggest that LC neurons might have basal ERK_1/2_ activity, which could regulate various types of ion channels involved in the regulation of the membrane potential of LC neurons. To minimize the nonspecific effects, we pretreated the slices for 2 hrs and continuously perfused them throughout the recording with U0126 or FR180204 so that a stable baseline could be obtained before the application of baclofen and CGP54626.In slices pretreated and perfused with U0126 or FR180204, the peak amplitude of I_GABABR_ showed no difference in U0126- (n = 9 cells) and FR180204- (n = 10 cells) treated slices compared to the peak amplitude of I_GABABR_ measured in control slices (Kruskal-Wallis test, p = 0.644 among the comparisons between control and ERK blocker groups) (Fig. 3A–D). In contrast, both drugs significantly accelerated the decline in I_GABABR_ compared with the control condition. The τ_50_ measured from I_GABABR_ recorded in the U0126-treated and FR180204-treated slices was 163 ± 21 s and 150 ± 21 s, respectively; both were significantly shorter than the measurements obtained using the control slice (Kruskal-Wallis test, p = 0.001 among the comparison between control and ERK blocker groups; p = 0.009 for control vs. U0126 and p = 0.002 for control vs. FR180204 using post hoc Dunn’s multiple comparisons test) (Fig. 3A–C,E). The I_Tonic_ revealed by the subsequent application of CGP54626 in slices pretreated with U0126 was −7.6 ± 4.0 pA; it was 0.9 ± 7.0 pA in slices pretreated with FR180204; no difference was observed in either case compared with the control (two-way repeated-measures ANOVA, sphericity assumed, F(1, 27) = 3.672, p = 0.066 for CGP effects; sphericity assumed, F (2, 27) = 1.337, p = 0.279 for the comparison among ERK blockers) (Fig. 3A−C,F).

Compared with baseline, the application of CGP54626 also increased the frequency of I_Osc_ in U0126- and FR180204-treated slices, with the extent of the increase being significantly less in FR180204-treated slices than that in the control slices (two-way repeated-measures ANOVA, sphericity assumed, F(1, 26) = 55.106, p = 0.000 for CGP effects; sphericity assumed, F (2, 26) = 6.255, p = 0.006 for the comparison among ERK blockers). Upon CGP54626 application, the frequency of I_Osc_ significantly increased from 0.24 ± 0.02 to 0.33 ± 0.3 Hz in U0126-treated slices (n = 9 cells, p = 0.025, post hoc Bonferroni test); however, the extent of the increase showed no difference compared to the control (p = 1, post hoc Bonferroni test). In FR180204-tretaed slices, the frequency of I_Osc_ significantly increased from 0.48 ± 0.06 to 0.57 ± 0.08 Hz (n = 9 cells, p = 0.032, post hoc Bonferroni test)and the extent of the increase was significantly less than that in the control (p = 0.001, post hoc Bonferroni test) (Fig. 3G). Again, the significant reduction in the extent of the increase in SFR upon CGP54626 application observed in FR180204-tretaed slices but not in U0126-treatred slices might be ascribed to the fact that FR180204 specifically targets ERK_1/2_, while U0126 targets the mitogen-activated protein kinase that acts upstream of ERK_1/2_.

### ERK_1/2_ activated by GABA_B_Rs is essential to sustain tonic inhibition of LC neurons

An interpretation of the above observations could be that the activation of GABA_B_Rs in LC neurons not only opened GIRK channels but also triggered ERK-dependent autoregulation of receptors to prevent the quick desensitization of GABA_B_Rs upon prolonged exposure to the agonist. To test this possibility, we examined the effects on tonic inhibition of LC neurons^7^ with prolonged exposure of GABA_B_Rs to baclofen at a saturating concentration with the inhibition of ERK activity. If the above interpretation is correct, the inhibition of ERK-dependent autoregulation would result in a reduced functionality of GABA_B_Rs, as indexed by a reduction in the tonic inhibition of LC neurons after prolonged agonist exposure. We found that a precise assessment of tonic inhibition by directly measuring GABA_B_R-mediated I_Tonic_ was difficult due to the high noise level imposed by the high I_Osc_ activity. This phenomenon might account for the lack of a significant difference in I_Tonic_ in the U0126- or FR180204-treated slices compared to the control (Fig. 3F).Accordingly, we examined the SFR of LC neurons to assess GABA_B_R-mediated tonic inhibition.

**Figure 5:**
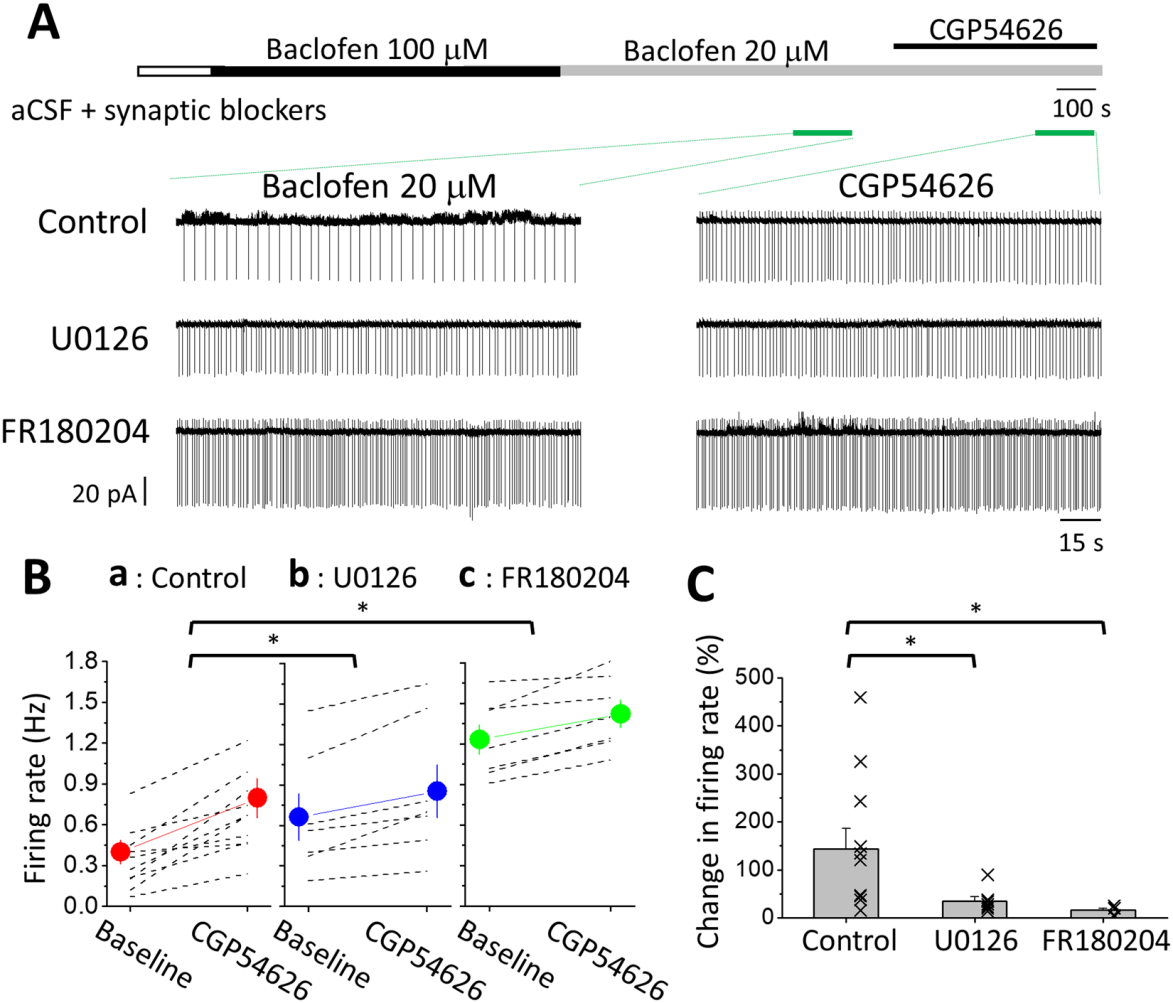
The inhibition of ERK_1/2_ attenuates GABA_B_R-mediated tonic inhibition of LC neurons. (**A**) The top bar shows the experimental protocol, and the bottom traces show representative episodes of recordings with baclofen (left panel) and CGP54626 (right panel) from an LC neuron in control slices (upper raw), U0126-treated (middle), and FR180204-treated slices (bottom raw). The recordings were performed using the cell-attached configuration to record spontaneous APs. (B & C) Summarized results show the change in the spontaneous firing rate upon CGP54626 application (B) and GABA_B_R-mediated tonic inhibition (C). Each dashed line (B) or cross (C) shows the results of an individual experiment; the circles (B) or bars (C) denote the mean, and capped lines denote the SEM. The asterisks indicate a significant difference compared to the control at p < 0.05 (*).

To examine the effects on tonic inhibition of LC neurons with prolonged exposure of GABA_B_Rs to baclofen with the inhibition of ERK activity, we perfused the slices with 100 μM baclofen, the minimum dosage for producing the saturation of GABA_B_R functionality in LC neurons^7^, for 15 min after obtaining a stable cell-attached recording from an LC neuron. The slices were then washed with 20 μM baclofen for an additional 15 min, followed by the co-administration of 20 μM baclofen and CGP54626 (Fig. 5A). Based on the dose-dependent relationship of I_GABABR_ induced by baclofen^7^, we estimated that 20 μM baclofen would produce 70% of the maximum GABA_B_R activation in LC neurons. Therefore, bathing the slices in 20 μM ambient baclofen could largely amplify tonic inhibition for easier observation. As seen (Fig. 5A,B), after a 15-minute period of pre-exposure to the agonist at a saturating concentration, a significant increase in the SFR upon CGP54626 application was observed in LC neurons bathed in 20 μM baclofen in the control slices (n = 10 cells, p = 0.000, Student’s paired t-test), the U0126-treated slices (n = 7 cells, p = 0.012, Student’s paired t-test) and in the FR180204-treated slices (n = 7 cells, p = 0.001, Student’s paired t-test). The GABA_B_R-mediated tonic inhibition under the condition was defined as:

$$(SF{R}_{Bac+CGP54626}\mbox{--}SF{R}_{Bac})\times 100 \% /SF{R}_{Bac}$$

where SFR_Bac_ and SFR_Bac+CGP54626_ are the SFRs recorded in 20 μM baclofen and 20 μM baclofen plus CGP54626, respectively. The calculated tonic inhibition was 147.3 ± 39.4% in the control (n = 10), which was significantly stronger than that in U0126-treated slices of 34.8 ± 8.9% (n = 7) and in FR180204-treated slices of 16.6 ± 3.2% (n = 7) (One-way ANOVA, p = 0.008 among the comparison between control and ERK blocker groups; p = 0.045 for control vs. U0126 and p = 0.013 for control vs. FR180204 using post hoc Bonferroni test) (Fig. 5C).

### ERK_1/2_ activated by GABA_B_R does not have an effect on pGABA_B2_R

**Figure 6:**
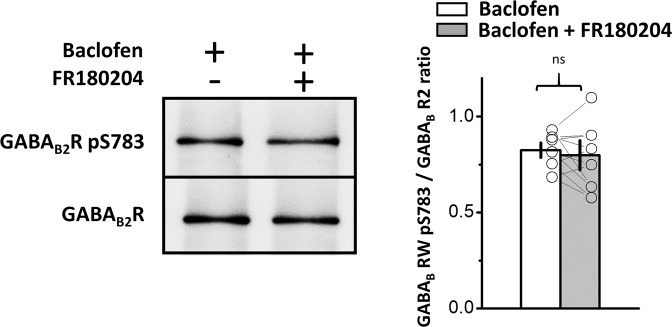
ERK_1/2_ activated by GABA_B_R does not have an effect on pGABA_B2_R. Left images show representative western blot analysis results for pGABA_B2_R in LC tissue punched from slices bathed in baclofen or in baclofen plus FR180204. The plot in the right panel summarizes the results. Each paired circle and line indicates the result of a single experiment; bars and vertical lines denote the mean and SEM, respectively. ns denotes no significance compared to the control.

Finally, we examined the possible regulatory site of ERK_1/2_ regulation of GABA_B_R functionality upon receptor activation. To this end, we compared the level of phosphorylated GABA_B2_ receptor subunit at the serine 783 (S783) site between LC tissue treated with baclofen and that treated with baclofen plus FR180204 using western blot analysis, as this regulatory site is shown to enhance GABA_B_R activation of GIRK^33^. If ERK_1/2_ regulated GABA_B_R functionality by phosphorylating the S783 site of the GABA_B2_ receptor subunit, the pGABA_B2_ receptor level should be significantly higher in baclofen-treated LC tissue than in the tissue treated with baclofen plus FR180204. However, we did not find a difference between the two groups in the level of phosphorylation of the GABA_B2_ receptor at the S783 site. Compared to the control (LC tissues from the baclofen-bathed slices), the pGABA_B2_R level was 96.3 ± 6.9% of the control in LC tissues from the slice bathed in baclofen plus FR180204 (Fig. 6) (n = 6, p = 0.651, Student’s paired t-test).

## Discussion

In this study, we provide biochemical and electrophysiological evidence showing that GABA_B_Rs can mediate the autoregulation of GABA_B_R-activated whole-cell current through the activation of ERK_1_ in LC neurons. Since ambient GABA in the LC is significantly higher during rapid eyemovement (REM)/non-REM sleep than during wakefulness^20,22^, ERK_1_-dependent autoregulation of GABA_B_R functionality could be a mechanism enabling the receptors to be continuously activated by the ambient GABA without undergoing severe desensitization, thereby providing a stable, tonic inhibition of LC neurons.

In many brain regions, including the LC, electron microscopic studies have shown that GABA_B_Rs are located predominantly close to neurotransmitter release sites at presynaptic terminals; in contrast, they are mainly located at peri-/extrasynaptic but not at synaptic active zones in postsynaptic neurons^4–7^. These locations of GABA_B_Rs imply that most of the receptors are not directly activated by synaptically released GABA in the synaptic cleft but by ambient GABA in peri-/extrasynaptic spaces. The concentration of ambient GABA critically depends on GABA spillover from the synaptic cleft, and the amount of GABA spillover is determined by the frequency and pattern of AP arrival at axonal terminals. It is also determined by the operation of GABA transporters located in the neuron and glia cell membrane^34–39^. GABA transporters not only serve in the reuptake of GABA in the synaptic cleft but also operate in a reverse mode that actually causes release but not reuptake of the neurotransmitter, a so-called nonvesicular release process, which would occur when high-frequency and repeated APs arrive at axonal terminals^40–42^. Accumulating reports have shown that ambient GABA generated by the abovementioned neuronal and glial activity could sufficiently and tonically activate peri-/extrasynaptic GABA_B_Rs at both presynaptic and postsynaptic sites, thereby exerting tonic inhibition for the control of transmitter release from presynaptic terminals and the spiking activity in postsynaptic neurons^7,9,14–16,34–36,39^. The regulation of ambient GABA by AP-dependent vesicular and nonvesicular release could modulate tonic inhibition of neurons that express peri-/extrasynaptic GABA_A_Rs and/or GABA_B_Rs, and the processes could be linked to the long-term regulation of brain function. LC neurons are well known to be involved in the regulation of the wakefulness–sleep cycle; they fire APs in a brain state-dependent manner, displaying active firing during wakefulness, a decreased discharge rate during non-REM sleep and silence during REM sleep^18,43^. Obviously, a mechanism that provides prolonged and stable inhibition of LC neurons could promote REM/non-REM sleep.

In addition to ambient GABA, the operation of GABA_B_R-mediated tonic inhibition would require a substantial and steady amount of GABA_B_Rs on the membrane. Nevertheless, many GPCRs undergo desensitization, a process in which a receptor reduces its response after prolonged exposure to the agonist, which helps to attenuate or terminate signal transduction to protect the target cells from overstimulation^25^. It is now well known that desensitization involves the phosphorylation of GPCRs by G protein-coupled receptor kinases (GRKs), which lead to the uncoupling of the receptors from G proteins. This process is followed by the internalization of GPCRs through the recruitment of arrestins that trigger clathrin-mediated endocytosis of the phosphorylated receptor^44^. Interestingly, a growing amount of evidence suggests that GABA_B_Rs, unlike many other GPCRs, are not the substrate for GRKs other than GRK4/5 and do not conform to the above-described agonist-induced internalization^45^. Nevertheless, GABA_B_Rs exhibit significant rates of constitutive endocytosis via clathrin-dependent and dynamin-dependent mechanisms under basal conditions, followed by the recycling of large numbers of GABA_B_Rs back to the plasma membrane to maintain steady-state cell surface numbers^45–48^. These processes perhaps reflect the long cell surface half-lives of GABA_B_Rs and make them suitable for mediating a stable and persistent effect such as tonic inhibition. For example, unlike neurokinin receptors, which undergo almost complete desensitization after a few seconds of exposure to substance-P in NAergic A7 neurons^29^, GABA_B_R desensitization progresses at a much slower rate in NAergic A7 neurons^9^ as well as in LC neurons. The regulation of the surface stability of the GABA_B_R number usually involves the phosphorylation of the receptor molecule per se. It has been shown that the phosphorylation of S892 of the GABA_B2_ receptor by protein kinase-A promotes cell surface stability of the GABA_B_R number^46^; in cultured hippocampal neurons, S867 in GABA_B1_ receptors is phosphorylated by Ca^2+^/calmodulin-dependent protein kinases downstream of NMDA receptor activation, which induces the internalization of GABA_B_Rs. The phosphorylation of the receptor molecule also regulates GABA_B_R signaling by altering the efficacy of receptor–effector coupling. The phosphorylation of S783 of theGABA_B2_ receptor by 5′AMP-dependent protein kinase (AMPK) has been shown to enhance GABA_B_R activation of GIRKs^33^. In addition to the promotion of cell surface stability of the receptor number, the phosphorylation of S892 of the GABA_B2_receptor by protein kinase-A facilitates receptor–effector coupling^49^ and slows KCTD12-induced desensitization of the GIRK current^50^. In addition to the receptor molecules, the phosphorylation of effectors, such as N-type Ca^2+^ channels, by tyrosine kinase can interact with regulators of G protein signaling (RGS) to form a regulatory complex that provides additional mechanism for regulation in the GABA_B_R-mediated currents^51^. Another mechanism reported to be involved in the regulation of GABA_B_R desensitization includesthe agonist-induced association of the receptors with GRK4/5, which results in GABA_B_R desensitization in a phosphorylation-independent manner. The interaction of GABA_B1_ and GABA_B2_ receptor subunits with NEM-sensitive fusion protein (NSF) primes the receptors for phosphorylation by protein kinase C (PKC) to uncouple them from G proteins. The association of GABA_B_Rs with various types of auxiliary proteins, such as potassium channel tetramerization domain-containing proteins (KCTDs), via the C-terminus of GABA_B2_ receptors can render the receptor complex competent for desensitization.

Our western blot analysis showed that baclofen could increase pERK_1_ levels in LC tissue, and this effect was not observed in LC tissue treated with CGP54626 to block GABA_B_Rs. These observations show that the baclofen-induced increase in pERK_1_ levels in the LC was GABA_B_R-dependent, consistent with the results of previous reports in the hippocampus and cerebellum^26–28^. In addition to the well-known effect on gating GIRKs, the results of these previous and present studies demonstrate an additional role of GABA_B_R activation in activating ERK in hippocampal CA1 pyramidal neurons, cerebellar granule neurons and LC neurons. By performing whole-cell recordings directly from LC neurons, we further observed that the inhibition of ERK_1/2_ with two selective blockers produced consistent effects on the activity induced by baclofen application in LC neurons. These results confirmed that the elevation of pERK_1_ levels by GABA_B_R activation occurred in LC neurons, although we could not completely rule out the possibility that the effects on LC neuron activity were secondary. We did not validate the link between GABA_B_R activation and the increased pERK_1_ level. However, it has been reported that the increase in pERK_1/2_ levels by GABA_B_R activation relies on a pertussis-toxin-sensitive G_i/o_ heterotrimeric protein-dependent pathway by releasing G_βγ_ in cultured cerebellar granule neurons^27^. Interestingly, this signaling pathway for ERK_1/2_ phosphorylation seems to be unique to GABA_B_Rs, as the activation of G_i/o_-coupled α_2_-adrenoceptors or G_i/o_-coupled 5HT_1A_ receptors in the adult mouse CA1 region did not result in ERK_1/2_ phosphorylation^26^. As shown in our electrophysiological experiments, the inhibition of ERK_1/2_ activity with two selective blockers accelerated the decay of I_GABABR_, and it is likely that the signaling mechanisms of GABA_B_R trafficking and/or desensitization may involve an ERK-dependent intermediate step that has not yet been identified. Therefore, the inhibition of the ERK-dependent intermediate step accelerated the decline of I_GABABR_. Our western blot analysis showed that the activation of ERK_1_ downstream of GABA_B_R activation did not have an effect on the level of phosphorylation of the GABA_B2_receptor at the serine 783 site. Activated ERK_1_ could regulate GABA_B_R functionality at other serine sites in GABA_B1_ and/orGABA_B2_ receptors, as previously described. Furthermore, it could be involved in the interaction between RGS and phosphorylated GIRKs by tyrosine kinase^51,52^, as it has been recently suggested that the GABA_B_R activates tyrosine kinase and downstream ERK_1/2_^53^. Apparently, the mechanisms underlying the regulation of GABA_B_R desensitization by ERK_1_ remain to be clarified.

In conclusion, here, we report that the activation of GABA_B_Rs with baclofen increases the phosphorylation of ERK_1_ in LC tissue and that the blockade of ERK_1/2_ activity with two selective blockers, U0126 and FR180204, accelerates the decay of I_GABABR_ induced by prolonged baclofen application in LC neurons. Furthermore, an increase in the spontaneous firing rate and a decrease in the tonic inhibition of LC neurons occurs after prolonged GABA_B_R activation by baclofen at a saturating concentration. These results suggest a new functional role of Gαi/ο upon GABA_B_R activation in mediating an ERK_1_-dependent autoregulation of the stability of GABA_B_R-activated whole-cell current in LC neurons, in addition to the well-known action of gating GIRKs with G_β,γ_ subunits. We argue that activated ERK_1_ signaling might help maintain a dynamic balance between the desensitization and recycling of receptors back to the membrane to sustain GABA_B_R-mediated tonic inhibition of LC neurons. However, it should be noted that postnatal day 8–10 rats were used in this study, and there is a possible weakness that the same phenomena may not be fully present in adulthood.

## Materials and Methods

### Animals

The use of animals in this study was approved by the Institutional Animal Care and Use Committee for National Taiwan University (permission #NTU103-EL-00076) and the Institutional Animal Care and Use Committee for Chung Shan Medical University(permission #1423), the guidelines of which comply with the “Codes for Experimental Use of Animals” of the Council of Agriculture of Taiwan based on the Animal Protection Law of Taiwan. Every effort was made to minimize the number of animals used and their suffering. Sprague-Dawley rat pups of both sexes were used and sacrificed for slice preparation on postnatal days 8–10 for the electrophysiological experiments and postnatal days 14–16 for the western blot analysis.

### Preparation of brainstem slices and electrophysiology

The animals were anesthetized with 5% isoflurane in O_2_ and decapitated, followed by rapid exposure of the brain and chilling with ice-cold aCSF consisting of the following (in mM): 119 NaCl, 2.5 KCl, 1.3 MgSO_4_, 26.2 NaHCO_3_, 1 NaH_2_PO_4_, 2.5 CaCl_2_, and 11 glucose, oxygenated with 95% O_2_ and 5% CO_2_, pH 7.4. Sagittal brainstem slices (300 μm) containing the LC were prepared using a vibroslicer (D.S.K. Super Microslicer Zero 1, Dosaka EM, Kyoto, Japan), maintained in a moist air-liquid (aCSF) interface chamber and allowed to recover for at least 90 minutes before being transferred to an immersion-type chamber mounted on an upright microscope (BX51WI, Olympus Optical Co., Ltd., Tokyo, Japan) for recording. Throughout the recording period, they were perfused at 2–3 ml/min with oxygenated aCSF containing synaptic blockers. The synaptic blockers contained 5 mM kynurenic acid, 100 μM picrotoxin and 1 μM strychnine to block glutamatergic, GABAergic and glycinergic synaptic transmission, respectively. The baseline described in the control slices refers to the recordings made under these conditions before administering baclofen and CGP54626. For recordings made in the U0126-treated slices and FR180204-treated slices, baseline refers to recording in the aCSF containing synaptic blockers, 0.1% DMSO and U0126 or FR180204.

Neurons were viewed using Nomarski optics. Patch pipettes were pulled from borosilicate glass tubing (1.5 mm outer diameter, 0.32 mm wall thickness; Warner Instruments Corp., Hamden, CT, USA) and had a resistance of approximately 3–5 MΩ when filled with the pipette solutions. To record the GABA_B_R-mediated current, the pipette solution consisted of the following (in mM): 131 K-gluconate, 20 KCl, 10 HEPES, 2 EGTA, 8 NaCl, 2 ATP, and 0.3 GTP; pH adjusted to 7.2 with KOH. Recordings were performed at 29–31 °C in the whole-cell or cell-attached configuration with a patch amplifier (Multiclamp 700 B; Axon Instruments Inc., Union City, CA, USA). For current-clamp recordings of whole-cell configuration, the bridge was balanced, and the recordings were accepted only if the recorded neuron had a membrane potential (V_m_) of at least −45 mV without applying a holding current and if the APs were able to overshoot 0 mV. For voltage-clamp recordings, the V_m_ was clamped to −70 mV unless specified. The serial resistance was monitored throughout the recording, and the data were discarded if the values varied by more than 20% of the original value, which was usually less than 20 MΩ. In a series of experiments, the cell-attached configuration was used. In the recording, the patch amplifier was set in voltage-clamp mode with the pipette voltage set to 0 mV (holding current = 0 pA) so that the recorded neurons were at their resting membrane potential. All signals were low-pass filtered at a corner frequency of 2 kHz and digitized at 10 kHz using the Micro 1401 interface running Signal software for episode-based capture and Spike2 software for continuous recording (Cambridge Electronic Design, Cambridge, UK). All data are presented as the mean±the standard error of the mean (SEM). For statistical comparisons, the normality of the data was first tested using the Shapiro-Wilk test. Student’s paired t-test and the nonparametric paired Wilcoxon-sign rank test were used for the comparison of data collected before and after drug application (Fig. 4, Fig.5B). One-way ANOVA was used for the comparison of I_GABABR_ parameters (Fig. 3D,E) and tonic inhibition (Fig. 5C) among the control, U0126 and FR180204 groups. Two-way repeated-measures ANOVA was used for the comparison of I_Tonic_ and oscillation rates (Fig. 3F,G) between baseline and CGP54626 application among the control, U0126 and FR180204 groups. The criterion for significance was *p* < 0.05.

All chemicals used to prepare the aCSF and pipette solution were from Merck (Frankfurt, Germany); baclofen, biocytin, carbenoxolone (CBX), kynurenic acid, picrotoxin, and strychnine were from Sigma (St. Louis, USA); and CGP54626 hydrochloride (CGP), U0126, and FR180204 were from Tocris-Cookson (Bristol, UK).

### Biocytin histochemistry and immunohistochemistry

In some experiments, 6.7 mM biocytin was included in the internal solution to fill the recorded neurons. The detailed procedures for viewing the biocytin-filled neurons and post hoc immunohistochemistry (IHC) for cell type identification of the recorded neurons have been described previously^29^. Briefly, after recording, the slices were fixed overnight at 4 °C in 4% paraformaldehyde (Merck) in 0.1 M phosphate buffer (PB), pH 7.4, and then subjected to biocytin histochemistry and IHC procedures without further sectioning. The slices were incubated for 1 hr at room temperature in phosphate-buffered saline (PBS) containing 0.03% Triton X-100 (PBST), 2% bovine serum albumin (BSA), and 10% normal goat serum (NGS), followed by incubation overnight at 4 °C in PBST containing a 1/2000 dilution of rabbit antibodies against TH (Merck Millipore, Darmstadt, Germany) and a 1/200 dilution of streptavidin Alexa Fluor 488 (Jackson ImmunoResearch, West Grove, PA, USA). After rinsing with PBST, they were incubated for 2 hrs with a 1/200 dilution goat anti-rabbit IgG antibodies conjugated to Alexa Fluor 594 (Jackson ImmunoResearch, West Grove, PA, USA) in PBST and then observed under a fluorescence microscope (Axioplan 2, Zeiss, Oberkochen, Germany) or a confocal microscope (LSM780, Zeiss Microsystems, Jena, Germany).

### Protein purification and western blot analysis

Brain slices were prepared using procedures described above and were maintained in the interface chamber and allowed to recover for at least 90 minutes. In each experiment, 5 or 6 rats were used for brain slice preparation. The slices from the left hemispheres were used as controls, and those from the right hemispheres were treated with a tested drug for comparison. After 15 min of drug or vehicle incubation at 30–32 °C, the LC was punched out using an 18 G needle with the tip blunted (Fig. 1A). The LC from the left hemispheres was pooled together in a test tube, as well as those from the right hemispheres in another tube. Then, the two tubes of LC tissue were simultaneously subjected to protein purification and western blotting under the same conditions. The above-described experiment was repeated 9 times in Fig. 1B1 (n = 9), 6 times in Fig. 1B2 (n = 6), and 6 times in Fig. 5 (n = 6). For protein purification, the samples were lysed in extraction buffer containing cell lysis buffer (Cell Signaling Technology), 1 μg/ml leupeptin, 1 μg/ml aprotinin, 2 mM PMSF, 0.5 mM Na_3_VO_4_, and 5 mM NaF and centrifuged at 16,100 × g for 30 min at 4 °C. The supernatant was then collected, and the protein concentration was determined using a BCA Protein Assay Kit (Pierce) with bovine serum albumin (BSA) as the standard. The samples (30 μg of protein) were separated by 8~10% SDS-PAGE and transferred to a nitrocellulose membrane (Bio-Rad Laboratories). The membrane was incubated with 5% BSA and 5% nonfat milk in Tris-Tween-buffered saline (TTBS) buffer containing 50 mM Tris-HCl (pH 7.5), 0.15 M NaCl, and 0.1% Tween 20 for 1 hr at room temperature, followed by an overnight incubation at 4 °C with primary antibodies in TTBS buffer. In Fig. 1B, the primary antibodies were rabbit polyclonal antibodies against p44/42 MAPK (ERK_1/2_) (1/1000, Cell Signaling Technology) or rabbit monoclonal antibodies against phospho-p44/42 MAPK (ERK_1/2_) (1/750, Cell Signaling Technology); in Fig. 5, the primary antibodies were rabbit monoclonal antibodies against the GABA_B2_ receptor (1/1000, Cell Signaling Technology) and rabbit polyclonal antibodies against phospho-S783 of the GABA_B2_ receptor (1/1000, Rockland Immunochemicals). The membrane was sequentially incubated for 1 hr at room temperature with biotinylated goat anti-rabbit IgG antibodies (Vector Laboratories), followed by avidin-biotinylated horseradish peroxidase (HRP) complex (Vector Laboratories) in TTBS. The bound antibodies were detected using an enhanced chemiluminescence (ECL) detection system (Fujifilm), and the intensities of the bands were quantified using the ImageGauge program (Fujifilm Software). For statistical comparisons, Student’s paired t-test was used, as the data all passed the normality test using the Shapiro-Wilk test (Fig. 1 and 6).
